# Invited review: Remediation strategies for mycotoxin control in feed

**DOI:** 10.1186/s40104-021-00661-4

**Published:** 2022-01-28

**Authors:** Meng Liu, Ling Zhao, Guoxin Gong, Lei Zhang, Lei Shi, Jiefan Dai, Yanming Han, Yuanyuan Wu, Mahmoud Mohamed Khalil, Lvhui Sun

**Affiliations:** 1 Hubei Hongshan Laboratory, College of Animal Science and Technology, Huazhong Agricultural University, Wuhan, 430070 Hubei China; 2 Department of Agriculture of Sichuan Province, Chengdu, 610041 China; 3 Trouw Nutrition, Amersfoort, The Netherlands; 4 Animal Production Department, Faculty of Agriculture, Benha University, Banha, 13736 Egypt

**Keywords:** Animal health, Feed, Mycotoxin, Performance, Remediation strategies

## Abstract

Mycotoxins are secondary metabolites of different species of fungi. Aflatoxin B_1_ (AFB_1_), deoxynivalenol (DON), zearalenone (ZEN) and fumonisin B_1_ (FB_1_) are the main mycotoxins contaminating animal feedstuffs. These mycotoxins can primarily induce hepatotoxicity, immunotoxicity, neurotoxicity and nephrotoxicity, consequently cause adverse effects on the health and performance of animals. Therefore, physical, chemical, biological and nutritional regulation approaches have been developed as primary strategies for the decontamination and detoxification of these mycotoxins in the feed industry. Meanwhile, each of these techniques has its drawbacks, including inefficient, costly, or impractically applied on large scale. This review summarized the advantages and disadvantages of the different remediation strategies, as well as updates of the research progress of these strategies for AFB_1_, DON, ZEN and FB_1_ control in the feed industry.

## Introduction

**Fig. 1:**
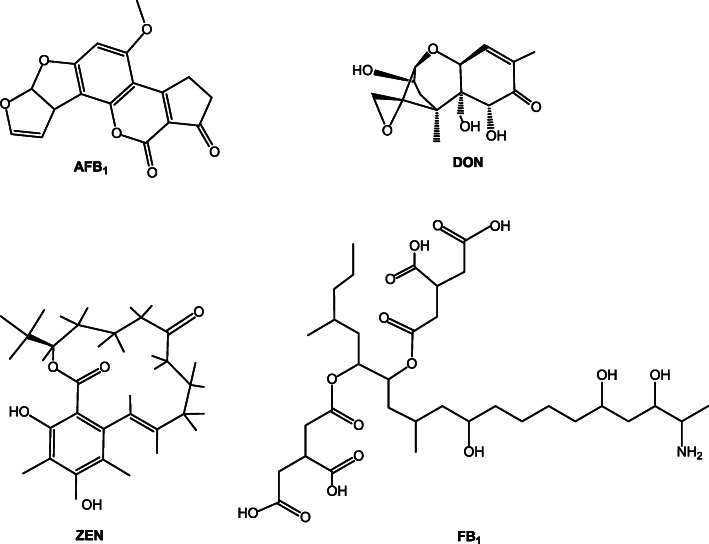
Structural diversity of AFB_1_, DON, ZEN and FB_1_. AFB_1_: Aflatoxin B_1_; DON: deoxynivalenol ; ZEN: zearalenone; FB_1_: fumonisin B_1_

Mycotoxins are secondary metabolites of various species of fungi that can cause chronic or acute toxicity in animals. Although over 500 mycotoxins have been identified, those of importance in feed safety are primarily produced by the five fungal genera *Aspergillus*, *Fusarium*, *Penicillium*, *Claviceps* and *Alternaria* [1–5]. Aflatoxin B_1_ (AFB_1_), deoxynivalenol (DON), zearalenone (ZEN) and fumonisin B_1_ (FB_1_) are well-known as the main mycotoxins contaminating animal feedstuffs, such as corn, barley, wheat, peanuts, peas, nuts, millet, forage, and their by-products [3–6]. The toxicity of these mycotoxins varies depending on their chemical structure (Fig. 1). The most toxic mycotoxin is AFB_1_, mainly produced by *Aspergillus*, which is classified as a Group one carcinogen [7]. It displays hepatotoxic, immunotoxic, mutagenic, carcinogenic and teratogenic characteristics in many animal species [8–11]. Notably, all of DON, ZEN and FB_1_ are primarily produced by *Fusarium* molds [5, 12]. DON, a type B trichothecene, can induce anorexia, vomiting, and endanger intestinal and immune functions in different animals by inhibiting the synthesis of nucleic acids and proteins [13–16]. ZEN has a similar structure to estrogen and thus competing with 17 β-estradiol for estrogen receptor binding, consequently leading to fertility and reproductive disorders in livestock [16–19]. FB_1_ is the most plentiful fumonisins, which can cause hepatotoxicity, neurotoxicity, nephrotoxicity, immunotoxicity, developmental toxicity and cancer in humans and animals [20].

Mycotoxins have been proven to have significant effects on animal health, performance, as well as quality and safety of products, this led to intensive studies over the past few decades on counteracting methods for mycotoxins control in feedstuffs and feed. Generally, physical, chemical, biological and nutritional regulation approaches have been developed as the main strategies for the detoxification of mycotoxins in the feed industry [21–26]. Nevertheless, many techniques have been proven to be inefficiency, costly, or impractically applied on large scale [21, 22]. The purpose of this review was to summarize the advantages and disadvantages of the various detoxification strategies, as well as update the research progress of these strategies for AFB_1_, DON, ZEN and FB_1_ control in the feed industry.

### The strategies of mycotoxin reduction and detoxification

#### Physical methods

Decontamination of mycotoxin by physical techniques mainly includes sorting and separation, washing, solvent extraction, heating, irradiation, and adsorption [27, 28]. The commonly used methods of physical detoxification of mycotoxins are summarized in Table 1.

**Table 1:** Summary of physical methods for mycotoxins decontamination^a^

Methods	Commonly used measures and reagents	Decontamination efficiency	References
Sorting and separation	Sieving, aspiration, gravity separation, photoelectric separation, image processing	Removed at least 51%, 63%, 93% of AFs, trichothecenes and fumonisins from the shelled white maize.	[27]
Washing and solvent extraction	Washing, solvent extraction (methanol, ethanol, hexane, acetonitrile, isopropanol and aqueous acetone etc.)	Removed aflatoxins, trichothecenes, ZEN and fumonisins by 51-72%, 64-69%, 2-61% and 73-74% from the grains through floating and washing with water.	[25, 27, 29]
Heating	High temperature, high voltage	Decomposited 78-88% of AFB_1_ in rice by cooking with pressure (0.10 MPa) at 160 °C for 20 min. Destroyed 90% of DON or ZEN in barely power at 220 °C in 11 or 85min. Reduced 80% FB_1_ while cooking rice at 100 °C for 10 min.	[30–32]
Irradiation	X-rays, γ-rays and electron beam, ultraviolet rays, infrared and microwave	Reduced 22.0-90.7% of AFB_1_ by irradiation. Decomposited 17.2-100% of DON by irradiation. Decontaminated 25.0-86.0% and 60.0-100% of ZEN by γ-rays and ultraviolet rays. FB_1_ was inactivated by 63.5-100%, 58.1% and 93.3% by γ-rays, electron beam and microwave in feedstuffs.	[33–41]

^a^*AFs* Aflatoxins, *AFB*_*1*_ Aflatoxin B_1_, *DON* deoxynivalenol, *ZEN* zearalenone, *FB*_*1*_ fumonisin B_1_

##### Sorting and separation

The mycotoxins are not uniformly distributed in grains, which mainly appeared in the moldy, broken and discolored parts [42, 43]. Meanwhile, the specific gravity of the mycotoxins-contaminated cereals is relatively lower than the normal ones. These characteristics enable sieving, aspiration, gravity separation, photoelectric separation, image processing techniques to be used to isolate the mycotoxins-contaminated feedstuffs [27, 44]. Specifically, Matumba et al. [27] reported that flotation, dehulling and hand sorting alone can remove at least 51%, 63%, 93% of aflatoxins (AFs), trichothecenes and fumonisins, respectively, from the shelled white maize, while 98% of these mycotoxins can be removed when combining three of these methods. However, these techniques are costly and only suitable for small-scale applications. Aspiration and gravity separation methods can reduce the DON in wheat, while it reduced the yield of harvested grain [21]. Additionally, near-infrared spectroscopy and optical visual sorting strategies can be used to detect the moldy maize and wheat kernels with more than 92% level of accuracy [22–28, 42–46].

##### Washing and solvent extraction

According to the water-soluble or fat-soluble properties of mycotoxin, it could be decontaminated by washing with water or extraction with organic solvent [47]. Floating and washing with water can remove AFs, trichothecenes, ZEN and fumonisins by 51-72%, 64-69%, 2-61% and 73-74%, respectively, from the grains [25, 27, 29]. Notably, floating and washing with a water solution consists of 10-30% NaCl, 30% sucrose, or 1 mol/L sodium carbonate can increase the removal rate of fumonisins from the corn and wheat [25, 48]. A combination of washing and hand sorting technologies together can reduce 84% of fumonisins [49]. The solvents, including methanol, ethanol, hexane, acetonitrile, isopropanol and aqueous acetone, are most commonly used for mycotoxin extraction. Previous studies showed that hexane-aqueous acetone-water (56%:42%:2%) and dimethyl ether can eliminate over 98% of AFs in oil crops [50, 51]. However, these methods have major disadvantages as they result in loss of nutrients, and costly due to drying and toxic extracts disposal, which limit their large-scale application.

##### Heating

Thermal treatment has been applied for the decontamination of mycotoxins in feed for many years. The efficiency of this method depends on the chemical structure and concentration of mycotoxins, temperature, duration, moisture content, pH and ionic concentration during the thermal treatment [52]. AFB_1_, DON, ZEN and FB_1_ are heat-stable compounds with decomposition temperatures more than 237, 175, 220, 150 °C, respectively [30, 53, 54], which makes it difficult to eliminate them by conventional thermal processing. Conventional hydrothermal treatment (cooking) with pressure (0.10 MPa) at 160 °C for 20 min can decompose AFB_1_ by 78-88% in rice [31], as well as pressure heating (0.10 MPa) at 120 °C for 4 h can degrade AFB_1_ by 95% in moist peanut powder [55]. Yumbe-Guevara et al. [30] reported that 90% of DON or ZEN in barley powder can be destroyed at 220 °C for 11 or 85 min. Frying chips at 190 °C for 15 min or drying rice from 150 to 200 °C for 40 min resulted in a loss of 67-70% of FB_1_, while cooking rice at 100 °C for 10 min reduced 80% of FB_1_ [32, 56]. Nevertheless, thermal treatments use an excessive amount of energy, also high temperature-induced Maillard reaction would reduce the nutritional values of feed ingredients. This led to a restriction in the application of heat treatments in the feed industry [33].

##### Irradiation

Irradiation might be a feasible technology for removing mycotoxins from the feed on an industrial scale. It can be classified into ionizing (x-rays, γ-rays and electron beam) and non-ionizing radiations (ultraviolet rays, infrared and microwave) [57, 58]. The action of irradiation on feedstuffs can induce physical, chemical and biological effects, which reduce or eliminate the mycotoxins [59, 60]. Specifically, AFB_1_ can be reduced by 43.0-87.8%, 65.7-71.5%, 22.0-100%, 90.7% by γ-rays, electron beam, ultraviolet rays and microwave, respectively, in different cereals [33–35]. DON can be decomposed by 37.0-82.4%, 17.2-56.3%, 83.4-100% by γ-rays, electron beam and ultraviolet rays, respectively, in feedstuffs [36–39]. ZEN can be decontaminated by 25.0-86.0% and 60.0-100% by γ-rays and ultraviolet rays, respectively, in grains [34, 36–38]. FB_1_ was inactivated by 63.5-100%, 58.1% and 93.3% by γ-rays, electron beam and microwave, respectively, in feedstuffs [35, 40, 41]. These different decomposition efficiencies of irradiation depend on the variation in the treatment condition, including doses and time of irradiation, the shape and composition of feedstuffs [61, 62]. Although irradiations can be considered as a potentially promising approach to decontaminate mycotoxins in feedstuffs, their safety issues such as mutagenesis that generates harmful microorganisms and damage the nutritional values of feedstuffs require a declaration and further studies.

##### Adsorption

Adsorption binders can form a complex with mycotoxins, thus prevent mycotoxins passage from the gastrointestinal tract into the blood and organs of animals. In the past decades, numerous binders from different origins have been investigated for their capacity to adsorb mycotoxins [52, 63]. Therefore, the adsorbent detoxification treatment is currently well understood and widely used to detoxify mycotoxins in the feed industry. In general, any ideal mycotoxin absorbent should possess these following properties, including high adsorption capacity against either range of mycotoxins (especially mycotoxins with low hydrophobicity), low non-specific binding to nutrients, as well as high safety, stability and palatability [52]. Table 2 shows lists of current patents related to adsorbing mycotoxin including AFB_1_, DON, ZEN and FB_1_ control in the feed.

**Table 2:** Summary of adsorbents with mycotoxins mitigation effects^a^

Adsorbent	Mycotoxins	Binding efficiency	Reference
Zeolite	AFB_1_	Decreased AFB_1_ residue in duck meat by 65% significantly and numerically decreased AFB_1_ residue in liver and egg.	[64]
Bentonite clay	AFB_1_	Decreased liver AFB_1_ residue by 41-87% when broilers fed AFB_1_ in diet.	[65]
Sodium bentonite	AFB_1_	Decreased liver AFB_1_ residue by 62.5% when broilers fed AFB_1_ in diet.	[66]
Modified maifanite	ZEN	Decreased ZEN residue in liver and muscle by 54.96% and 42.41% respectively at the dose of 1% when pig fed 1.11 mg/kg AFB_1_ in diet.	[67]
Bentonite or montmorillonite	AFB_1_, ZEN	Decreased rumen concentration of AFB_1_ and ZEN, decreased AFM_1_ in milk and ZEN in feces.	[68]
Organo-clay composites	AFB_1_	Decreased AFB_1_ concentrations in liver, kidney and plasma significantly in chickens.	[69]
Tri-octahedral bentonite	DON, ZEN	Adsorbed more than 90% of ZEN and FB_1_ while the adsorption dose up to 0.20%, w/v.	[70]
Pillared montmorillonite	DON	Adsorbed 14.7-23.4% and 21.8-27.4% of DON at at pH 2.0 and pH 6.8.	[71]
Nonionic surfactant octylphenol polyoxyethylene ether modified montmorillonites	AFB_1_, ZEN	The adsorption capacities of modified montmorillonites to AFB_1_ and ZEN increased up to 2.78 and 8.54 mg/g respectively from 0.51 and 0.00 mg/g by the raw montmorillonite.	[72]
Hydrated sodium calcium alumino silicate	AFB_1_, FB_1_	Adsorbed AFB_1_and FB_1_ in an aqueous solution, and the adsorption ratio ranged from 95.3% to 99.1% and 84.7% to 92.4%, respectively.	[73]
Modified Hydrated sodium calcium alumino silicate	DON	Reduced the toxicity of DON in weaning piglets.	[16]
Esterified glucomannan	AFs, ZEN, DON	Adsorbed 95%, 80% and 12% of aflatoxin, ZEN and DON.	[73, 74]
Inactivated yeast cell wall and low Yeast fermenting volatile organic compound	AFs, DON	Decreased AFs and DON synthesis by 82% and 93% respectively.	[75]
Distillers' wet grain, distillers' dried grains and distillers' dried grain with solubles	DON, ZEN	Adsorbed 48.9% and 67.9% of DON and ZEN (1 ppm each) using 5 g/L of micronized (20 mkm) yeast mass at 37 °C for 1h.	[76]
Yeast cell wall extract	ZEN	Adsorbed 40% of the total ZEN content in the intestines in monogastric animals.	[77, 78]
Activated charcoal	AFB_1_, ZEN	Reduced the toxicity of AFB_1_ on broilers and decreased the absorption rate of ZEN in small intestine from 32% to 5% when adding 2%.	[79, 80]
Cholestyramine	ZEN	Decreased the absorption rate of ZEN in small intestine from 32% to 16%.	[80]
Magnetic carbon nanocomposites	AFB_1_	Adsorbed nearly 90% of AFB_1_ within 180 min at pH 7.0.	[81]
Cross-lined chitosan polymers	AFB_1_, ZEN, FB_1_, DON	Adsorbed 73% AFB_1_, 94% ZEN and 99% FB_1_, but the adsorption ratio of DON less than 30%.	[82]
Polyvinylpyrrolidone	ZEN	Adsorbed 2.1 mg/g of ZEN.	[83]
*Lactobacillus casei*	AFB_1_	Reduced the absorption of aflatoxin in the intestinal tract significantly.	[84]
*Lactobacillus plantarum* F22	AFB_1_	Adsorbed 56.8% of AFB_1_.	[85]
*Lactobacillus plantarum* B7	FB_1_	Adsorbed 52.9% of FB_1_.	[86]
*Lactobacillus pentosus* X8	FB_1_	Adsorbed 58% of FB_1_.	[86]

^a^*AFs* Aflatoxins, *AFB*_*1*_ Aflatoxin B_1_, *DON* deoxynivalenol, ZEN: zearalenone; FB_1_: fumonisin B_1_

Aluminosilicate minerals, as the largest class of mycotoxin adsorbents, are the most widely applied and studied minerals in the decontamination of mycotoxin. Such adsorption binders mainly include bentonite, montmorillonite, zeolite, hydrated sodium calcium aluminosilicate, kaolin, illite, etc. [63]. The binding efficacy of mineral adsorbents is associated with the structures of both the binders and the mycotoxins. The binding efficiency depends significantly on the surface area, charge distribution and pore size of adsorption binders and the charge distribution, polarity and shape of the mycotoxins [52]. Some mycotoxins such as AFs have an ionic charge, thus clay minerals such as bentonite, illite, zeolite and kaolin are effective at removing them from the feed with more than 90% efficiency [87]. Numerous studies reported that zeolite, bentonite clay and sodium bentonite decreased AFB_1_ residues in the liver by 41-87% and numerically decreased AFB_1_ residue in the meat and egg when broilers or ducks fed AFB_1_ contaminated diet [64–66]. Chen et al. [67] reported that ZEN residue in liver and muscle of pigs were decreased by 55.0% and 42.4%, respectively, when supplemented with 1.0% modified maifanite in diet included 1.11 mg/kg ZEN. In ruminant feed, bentonite or montmorillonite decreased rumen concentration of AFB_1_ and ZEN and also decreased AFM_1_ in the milk and ZEN in the feces in goats [68]. Tzou et al. [69] prepared organo-clay composites by mixing bentonite-enriched clay with nonionic surfactants (Brij 30 and Igepal CO-890) and added organo-clay composites to feed. After chickens had consumed amended feed for 11 weeks, AFB_1_ concentrations in the liver, kidney, and plasma were significantly lower than the AFB_1_ control dietary treatment. Although many aluminosilicate adsorbents can adsorb strongly polar toxins, such as AFB_1_, FB_1_, etc. as supported by many studies, they appear to be ineffective at absorbing other non-aflatoxin mycotoxins including DON and ZEN [88, 89]. Bentonites have been considered as promising adsorbents for high-efficient removal of mycotoxins from the animal feed as they are eco-friendly, low-cost and highly efficient in adsorption of mycotoxins, modifying clays also could help to increase their adsorptive ability to non-polar mycotoxins [90–92]. To date, only one di-octahedral bentonite (1m588) was authorized as an anti-aflatoxin additive by the EU Regulation in 2009 [93]. Vila-Donat et al. [70] reported that tri-octahedral bentonite could adsorb more than 90% of ZEN and FB_1_ while the adsorption dose up to 0.20% (w/v). Nonionic surfactant octylphenol polyoxyethylene ether and modified montmorillonites, as mycotoxins adsorbent, were used for adsorption of AFB_1_ and weak polar ZEN in both single and binary-contaminate systems by simulating the conditions of the gastrointestinal tract. Modified montmorillonites increased the adsorption capacities to AFB_1_ from 0.51 mg/g of raw montmorillonite to 2.78 mg/g and ZEN from 0.00 mg/g of raw montmorillonite to 8.54 mg/g [72]. Adsorption of DON by pillared montmorillonite modified with aluminum, iron and titanium was investigated using UPLC-MSMS (at pH 2.0 and 6.8) and the results demonstrated that the adsorption ratios were 14.7-23.4% at pH 2.0 and 21.8-27.4% at pH 6.8 [71]. The commercially hydrated sodium calcium aluminosilicate has an excellent capability of adsorbing AFB_1_ and FB_1_ in an aqueous solution, and the adsorption ratio ranged from 95.3-99.1% and 84.7-92.4% of the available AFB_1_ and FB_1_, respectively [73]. Mineral adsorbents have been modified with quaternary long-chain alkyl/aryl amines to improve the adsorption of non-aflatoxin mycotoxins [74]. The binder Amdetox™ is mainly comprised of hydrated sodium calcium aluminosilicate that has been modified by cetylpyridinium chloride and intercalation with β-glucan [94]; these modifications increase the surface area of hydrated sodium calcium aluminosilicate, which maximizes the binding of mycotoxins with minimal adsorption of nutrients. Zhang et al. [16] reported that a modified hydrated sodium calcium aluminosilicate adsorbent could reduce the toxicity of DON in weaning piglets [16]. Furthermore, it must be noted that these adsorbents can adsorb micronutrients and have negative effects on the bioavailability of trace minerals and vitamins.

Second generation adsorbents have been developed originating from the cell wall component of microorganisms. Glucomannan is a common adsorbent that cannot be used by gut microbes and strongly adsorbed toxic substances and harmful pathogenic bacteria in animals. Mycotoxins can be adsorbed by esterified glucomannan, which is a kind of broad-spectrum mycotoxin adsorbent with an effective binding ability for AFs, ZEN, FBs and DON by 95%, 75%, 59% and 12%, respectively [73, 74]. Esterified glucomannan has been proved to improve the adverse consequences of mycotoxins on the performance, immunity, blood haematological and biochemical indices of chickens [70, 76, 78, 94]. The β-*D*-glucan chains of yeast cell walls have been demonstrated to effectively inactivate ZEN [77, 95]. Zeidan et al. [75] reported that inactivated yeast cell walls and low yeast fermenting (*L. thermotolerans*) volatile organic compounds could decrease AFs and DON synthesis by 82% and 93%, respectively, in vitro. A combination of mineral clay and yeast cell walls showed a considerably enhanced binding capacity of AFs, ZEN and fumonisins in an in vitro study; however, the adsorption abilities toward DON, ochratoxin A and T-2 toxin were low (< 60%) [96]. The yeast biomass obtained from distillers’ wet grain, distillers’ dried grains and distillers’ dried grain with solubles have the ability to bind various mycotoxins and adsorbed 48.9% and 67.9% of DON and ZEN (1.0 mg/kg each), respectively, using 5.0 g/L micronized yeast mass at 37 °C for 1 h [76]. In addition, the yeast cell walls extract adsorbed ZEN in the gastrointestinal tracts of monogastrics [77] and was able to adsorb 40% of the total ZEN contents in the intestines [78].

Activated charcoal, as a general adsorbent, has a large surface area and excellent adsorption capabilities in aqueous environments. Activated charcoal has demonstrated the ability to reduce AFs, ZEN, DON due to its porous structure in several studies [97, 98]. The partial protection induced by activated charcoal in lowering mycotoxin residues in the liver of broilers has been observed previously [65, 99]. The addition of 0.1% activated carbon to feed containing 10 mg/kg AFB_1_ was able to reduce the detrimental effects of AFB_1_ on broilers [79]. Avantaggiato et al. [80] found that the absorption rate of ZEN in the small intestine decreased from 32% to 5% when activated carbon was added at 2.0% in an in vitro gastrointestinal model. Cholestyramine is an anion exchange resin. The addition of cholestyramine decreased the absorption rate of ZEN in the small intestine from 32% to 16% using a laboratory model that mimics the metabolic processes of the gastrointestinal tract of healthy pigs [80]. Polyvinylpyrrolidone has good adsorption and selectivity. In vitro adsorption experiments showed that polyvinylpyrrolidone could adsorb 0.3 mg/g of ZEN, and the adsorption capacity of modified polyvinylpyrrolidone could be increased to adsorb 2.1 mg/g of ZEN [83]. Durian peel is an agricultural waste that is widely used for organic and inorganic pollutant adsorption. Adunphatcharaphon et al. [100] reported that the acid-treated durian peel adsorbed 98.4% of AFB_1_, 98.4% of ZEN, 86.1% of FB_1_ and 2.0% of DON through its larger surface area and a surface charge modification.

Numerous studies have suggested that removal of mycotoxins with magnetic materials is effective and these are promising adsorbents in the feed industry. Magro et al. [101] reported that the adsorbent-mycotoxin complex was characterized and was structurally and magnetically well conserved [101]. Magnetic carbon nanocomposites produced by maize wastes were used for the removal of AFB_1_, and the adsorption ratio was nearly 90% within 180 min at pH 7.0 [81]. In addition, cross-linked chitosan polymers as generic adsorbents for simultaneous adsorption could adsorb multiple mycotoxins. Cross-linked chitosan-glutaraldehyde complex presented high adsorption capability for AFB_1_ (73%), ZEN (94%) and FB_1_ (99%), but no obvious adsorption for DON and T-2 toxin (< 30%) [82]. Some volatile bioactive compounds have proved to be effective in inhibiting mould growth and reducing mycotoxin accumulation.

The principle of microbial adsorbent detoxification is that the bacterium adsorbs mycotoxins to form a complex and then excretes it together with the toxins, thus reducing the hazard [102]. Lactic acid bacteria and yeast are the most studied microbial adsorbents. *Lactobacillus casei* can significantly reduce the absorption of aflatoxin in the intestinal tract [84]. Zeng et al. [85] reported that *Lactobacillus plantarum* F22 had a strong adsorption capacity on AFB_1_ and the adsorption rate could reach 56.8%. *Lactobacillus plantarum* B7 and *Lactobacillus pentosus* X8 can remove 52.9% and 58.0% of FB_1_ [86]. Halttunen et al. [103] compared the adsorption effect of multiple lactic acid bacteria on aflatoxin and they found that a composite agent consisting of multiple lactic acid bacteria was more effective than a single strain.

#### Chemical methods

Chemical techniques can destroy the structure of the mycotoxins, which generate mildly toxic or nontoxic products. Decontamination of mycotoxin by chemical techniques primarily includes alkaline and ozone treatments, as well as other chemical agent treatments [104, 105]. The commonly used methods of chemical detoxification of mycotoxins are summarized in Table 3.

**Table 3:** Summary of physical methods for mycotoxins detoxification^a^.

Methods	Measures and reagents	Detoxification efficiency	Reference
Alkaline treatment	Ammonia, sodium hydroxide, potassium hydroxide and sodium carbonate etc.	Removed 95% of AFB_1_ in various cereals by ammoniation and hydroxide salts treatments.	[106–111]
		Reduced DON by 83.9-100% in different feedstuffs through sodium carbonate and hydroxide salts treatments.	
Ozone treatment	Ozone, hydrogen peroxide, chlorine, sodium and calcium hypochlorite etc.	Reduced 92-95%, 91% and 78% of AFBs in corn, cottonseed and peanut meal respectively by ozone. DON can be reduced 70-90% in corn and 20-80% in wheat by ozone. The degradation of ZEN in corn can reach 90.7% through the ozone treatment with 100 mg/L ozone for 180 min.	[112–119]

^a^*AFB*_*1*_ Aflatoxin B_1_, *DON* deoxynivalenol, *ZEN* zearalenone, *FB*_*1*_ fumonisin B_1_

##### Alkaline treatment

Alkaline chemicals, including ammonia, sodium hydroxide, potassium hydroxide and sodium carbonate, etc., have been used for the destruction of various mycotoxins in the moldy feedstuffs [104, 105]. The lactone ring structure of AFB_1_ can be opened by base hydrolysis to produce coumarin sodium salt and then further be eliminated by washing with water [120]. Ammoniation and hydroxide salts treatments are the common approach that has been used to remove AFB_1_ from feed ingredients, with more than 95% removal rate in various cereals [107–110]. An epoxide at C-12 and C-13, essential for the toxicity of DON, can be destructed under alkaline conditions [28]. Sodium carbonate and hydroxide salts treatments can reduce DON by 83.9-100% in different feedstuffs [111, 112]. Although these treatments could nearly reduce the complete concentration of mycotoxins, the possible transformation of mycotoxins to other forms such as masked mycotoxins, along with the harmful side effects on the environment and food (changes in nutritional quality, texture, or flavor), the quality and safety assessments of chemically treated products are necessary [104, 105].

##### Ozone treatment

Mycotoxin oxidizing agent treatment is an effective detoxification method through changing the molecular structure of mycotoxins. The oxidizers commonly used are ozone, hydrogen peroxide, sodium and calcium hypochlorite, chlorine and other oxidizers [106, 121]. AFs, DON, ZEN and FB_1_ have been shown to be effectively degraded by ozone [122–124]. Agriopoulou et al. [125] has found that ozone has the ability to degrade AFs (AFB_1_, AFB_2_, AFG_1_ and AFG_2_). Trombete et al. [126] reported that ozone concentration, form and exposure time influenced positively the reduction of DON, AFs and fungal count. AFs can be reduced by 92-95% in corn and by 91% or 78% in cottonseed or peanut meal, respectively, by ozone [113, 127, 128]. DON can be reduced by 70-90% in corn and by 20-80% in wheat by ozone [112, 114–116]. The degradation of ZEN in corn can reach 90.7% through the ozone treatment with 100 mg/L ozone for 180 min [117]. Furthermore, there are other oxidizing agents such as sodium hypochlorite and hydrogen peroxide that can effectively degrade mycotoxins [118, 119, 129, 130].

Although the ozone treatment can result in a complete reduction in the mycotoxin concentration, it can cause changes in the physical and chemical composition of the feed, such as changes in starch structure, lipid oxidation, protein denaturation, color change and processing properties [106, 113, 126]. Moreover, these treatments may produce some harmful chemicals to the health of animals [106, 113, 126].

#### Biological methods

Although many physical and chemical decontamination strategies have been developed to reduce or eliminate mycotoxins in feed ingredients or complete feed, few techniques met the requirements of practical application owing to their limitation of binding efficiency, bio-safety or cost-effectiveness. Therefore, as a promising strategy, biodegradation of mycotoxin by microorganism or enzymes attracted the attention of scientists [131–133]. The biological strategies that have been developed for the biodegradation of AFB_1_, DON, ZEN and FB_1_ in the feed are presented in Table 4.

**Table 4:** Biological biotransformation approaches by microorganisms for the detoxification of mycotoxins^a^.

Mycotoxins	Microorganisms	Biotransformation efficiency	Reference
AFB_1_	*Aspergillus niger* FS10	98.65%	[133]
	*Aspergillus niger* RAF106	88.59%	[132]
	*Stenotrophomonas* sp. CW117	100.00%	[134]
	*S. cerevisiae* ŁOCK 0119	69.00%	[131]
	*Escherichia coli* CG1061	93.70%	[135]
	*Bacillus velezensis* DY3108	91.50%	[136]
	*Bacillus subtilis* JSW-1	67.20%	[137]
	*Bacillus shackletonii* L7	92.10%	[138]
	*Bacillus licheniformis* CFR1	94.70%	[139]
	*Pseudomonas putida*	90.00%	[140]
	*Bacillus subtilis* UTBSP1	95.00%	[141]
DON	*Bacterial consortium* C20	74.29%	[142]
	*Bacillus subtilis* ASAG 216	81.10%	[143]
	*Devosia insulae* A16	88.00%	[144]
	*Pseudomonas* sp. Y1 and *Lysobacter* sp. S1	100.00%	[145]
	*Eggerthella* sp. DII-9	100.00%	[146]
	*Aspergillus* (NJA-1)	94.40%	[147]
	Bacterial isolates LS100 & SS3	100.00%	[148]
	Bacterial strain BBSH 797	-	[149]
	Strain E3-39	100.00%	[150]
ZEN	*Bacillus subtilis*	100.00%	[151]
	*Bacillus natto*	87.00%	[151]
	*Bacillus pumilus* ES-21	95.70%	[152]
	*Bacillus amyloliquefaciens* ZDS-1	95.70%	[153]
	*Bacillus subtilis* ANSB01G	88.65%	[154]
FB_1_	Bacterial consortium SAAS79	100.00%	[155]
	Strain NCB 1492	100.00%	[156]
	*Saccharomyces cerevisiae* IS1/1 and SC82	22%-50%	[157]
	*Bacillus* spp. S9, S10 and S69	43%-83%	[158]

^a^*AFB*_*1*_ Aflatoxin B_1_, *DON* deoxynivalenol, *ZEN* zearalenone, *FB*_*1*_ fumonisin B_1_

-means the biotransformation efficiency did not reported

##### Microorganisms with detoxification activities

Biology-based detoxification methods are widely recognized as specific, efficient and environment-friendly. The nutritive and sensory characteristics like color and flavor are reserved without involving harmful chemicals. Screening and isolating naturally existing microorganisms that show biotransformation capabilities against specific mycotoxins have been a popular strategy. Mycotoxin biodegradation technology is the process by which the toxic group of the mycotoxin molecules is broken down and destroyed by the secondary metabolites produced by microorganisms or their secreted intracellular and extracellular enzymes, while producing non-toxic or less toxic degradation products.

A number of different fungal have been shown to detoxify AFB_1_. Fungal strains such as *S. cerevisiae *ŁOCK 0119 has been shown to degrade AFB_1_ at levels of 69.0% [131]. Similarly, some studies reported that the ability of various *Aspergillus* strains such as *A. niger *FS10 and *A. niger* RAF106 have shown the ability to degrade AFB_1_ to levels between 88.6% and 98.7% [132, 133]. Bacteria degraded AFs mainly by secreting extracellular enzymes. Some strains of *Nocardia corynebacterioides*, *Flavobacterium aurantiacum* and *Bacillus* have been shown to degrade AFB_1_. Smiley and Draughon reported that the degradation efficiency of AFB_1_ by *Nocardia corynebacterioides* reached 74.5% in 24 h [159]. *Flavobacterium aurantiacum* could degrade AFB_1_ efficiently and its crude protein extract could degrade 74.5% of AFB_1_ [160, 161]. *Bacillus* is an important class of bacteria capable of degrading AFB_1_. Farzaneh et al. [141] isolated *Bacillus subtilis* UTBSP1 from Iranian pistachio nut and the degradation rate of AFB_1_ reached 78.4-95.0%. *Bacillus subtilis* ANSB060 isolated from the fish intestine could degrade 81.5% of AFB_1_ within 72 h [162]. In addition, other *Bacillus* such as *Bacillus licheniformis* CFR1, *Bacillus velezensis* DY3108, *Bacillus subtilis* JSW-1 and *Bacillus shackletonii* L7 have been able to degrade AFB_1_ to levels between 67.2-94.7% [136–139]. Other bacteria such as *Pseudomonas putida*, *Escherichia coli* CG1061 and *Stenotrophomonas* sp. CW117 also showed very efficient biodegradation rates up to 90% or more for AFB_1_ [134, 135, 140].

*Devosia insulae* A16, Strain E3-39, *Bacterial consortium* C20, *Pseudomonas* sp. Y1 and *Lysobacter* sp. S1 isolated from soil samples can convert DON to 3-keto-DON or 3-epi-DON, a less toxic derivative [142, 144, 145, 150]. Several studies have revealed that these strains resulted in 74-100% reduction of DON [142, 144, 145, 150]. From a different point of view, *Bacterial isolates *LS100 and SS3, Bacterial strain BBSH 797 and *Eggerthella *sp. DII-9 presented a high biotransformation activity of converting DON to diepoxy-deoxynivalenol [146, 148, 149]. Strains isolated from the intestine of donkeys and soil samples, namely *Bacillus subtilis *ASAG 216 and *Aspergillus *(NJA-1) have shown to decrease DON concentration by 81.1% and 94.4% [143, 147].

Microorganisms metabolize ZEN mainly through conversion or degradation to α-zearalenol, β-zearalenol, sulfate and other secondary metabolites with low or non-toxicity. *Bacillus natto* and *Bacillus subtilis* strains were shown to remove ZEN from the liquid medium: more than 75% ZEN could be biodegraded after incubation. In another study, up to 99% of ZEN was degraded by *B. subtilis* strain [151]. Lei et al. [154] isolated *Bacillus subtilis *ANSB01G from broiler intestinal chyme, and the degradation rate of ZEN by this strain in a liquid medium, natural mold corn, distillers' dried grain with solubles and a complete pig feed were 88.7%, 84.6%, 66.3% and 83.0%, respectively. *Bacillus pumilus* ES-21 and *Bacillus amyloliquefaciens* ZDS-1, isolated from soil samples, showed 95.7% reduction of ZEN [152, 153].

Some fungal and bacterial microorganisms have been reported to be able to degrade fumonisins. Styriak et al. [157] screened two strains of preserved yeast from the laboratory that were able to significantly degrade fumonisins in the culture medium. One is *Saccharomyces cerevisiae *IS1/1, which can degrade 45% of FB_1_ and 50% of the mixture FB_1_ and FB_2_ in the culture medium, the other one is *Saccharomyces cerevisiae* SC82, which also degrade FB_1_ and the mixture FB_1_ and FB_2_, the degradation rates were 22% and 25%, respectively [157]. Camilo et al. [158] screened three strains such as *Bacillus *spp. S9, S10 and S69, that degraded 43%, 48% and 83% FB_1_, respectively. Strain NCB 1492, isolated from soil samples, can completely degrade FB_1_ under 25°C, after 24 h [156]. Notably, another study reported that the degradation rate of FB_1_ by *Bacterial consortium* SAAS79 can reach 100% [155].

##### The usage of catabolizing enzymes

Although some microorganisms are highly active in biodegrading mycotoxins, some of them might secrete harmful metabolites or cannot survive in the gastrointestinal tract of the animals [163, 164]. Therefore, screening the enzymes from these microorganisms might be the promising strategy to solve the issues. Recently, there are many researches that have focused on the isolation of the enzymes that can biodegrade AFB_1_, DON, ZEN and FB_1_. The enzymes for the biodegradation of AFB_1_, DON, ZEN and FB_1_ in the feed are presented in Table 5.

**Table 5:** The usage of degrading enzymes for the detoxification of mycotoxins^a^

Mycotoxins	Degrading enzyme	Origin	Reference
AFB_1_	Bacillus aflatoxin-degrading enzyme	*Bacillus shackletonii* L7	[138]
	Manganese peroxidase	*Pleurotus ostreatus*	[165]
	Aflatoxin-Oxidase	*Armillariella tabescens*	[163]
	Myxobacteria aflatoxin degradation enzyme	*Myxococcus fulvus* ANSM068	[166]
	Laccase	White rot fungi	[167]
DON	Manganese peroxidase and Lignin peroxidase	Spent Mushroom Substrate	[168]
	Quinone-dependent dehydrogenase, NADPH-dependent aldo/keto reductases	*Devosia* sp. D6-9	[169]
	Aldo-keto reductase DepA/DepB	*Devosia mutans* 17-2-E-8	[170]
	Peroxidase	Rice bran	[171]
	Cytochrome P450 system	*Sphingomonas* sp. strain KSM1	[172]
ZEN	ZEN-specific lactonohydrolase	Recombinant enzymes	[173]
	A fusion enzyme by combining ZEN-specific lactonohydrolase and carboxypeptidase	*Clonostachys rosea* strain IFO7063 and *Bacillus amyloliquefaciens* strain ASAG1	[174]
FB_1_	Fumonisin carboxylesterase FumD	Recombinant enzymes	[175]

^a^*AFB*_*1*_ Aflatoxin B_1_, *DON* deoxynivalenol, *ZEN* zearalenone, *FB*_*1*_ fumonisin B_1_

The main fungal enzymes known to have degradation activity against AFB_1_ are laccase and oxidase [163]. The enzyme for AFB_1_ detoxification designated as aflatoxin-detoxifizyme was reported [164]. The gene was identified and cloned from an *Armillariella tabescens*. The recombinant aflatoxin-detoxifizyme was able to detoxify AFB_1_ and significantly reduce its mutagenic effects. Manganese peroxidase (1.5 U/mL) can degrade 90% AFB_1_ after 48 h of reaction [165]. Alberts et al. [167] recombinantly expressed the laccase gene by gene cloning and its degradation rate of AFB_1_ was 55%. Bacillus aflatoxin-degrading enzyme and myxobacteria aflatoxin degradation enzyme secreted by *Bacillus shackletonii* L7 and *Myxococcus fulvus* ANSM068 are also efficient in degrading AFB_1_ [138, 166].

Although there are early reports on an NADH-dependent bacterial cytochrome P450 system that transforms DON into 16-hydroxy-DON, no efficient DON biotransformation enzymes are patented yet [172]. Peroxidase such as manganese peroxidase and lignin peroxidase showed the potential for significant DON degradation [168, 171]. Aldo-keto reductase DepA and DepB can transfer DON to 3-keto-DON and 3-epi-DON which have lower toxicity than DON [170]. A quinone-dependent dehydrogenase and two NADPH-dependent aldo/keto reductases (AKR13B2 and AKR6D1) can detoxify deoxynivalenol in wheat via epimerization in a Devosia strain [169].

Laccases are copper-containing oxidases have high potential in degrading the heat-stable mycotoxin ZEN, which involved in many industrial application [176, 177]. A novel ZEN-specific lactonohydrolase was developed previously as a producer of different hydrolytic enzymes for feed biorefinery. The recombinant ZEN-specific lactonohydrolase secreted by the transformed fungal clones into the culture liquid was shown to remove ZEN [173]. A recombinant fusion enzyme by combining two single genes named ZEN-specific lactonohydrolase and carboxypeptidase have demonstrated that can completely degrade ZEN to the non-toxic product in 2 h at an optimum pH of 7 and a temperature of 35 °C [174].

The fumonisin carboxylesterase FumD can degrade FB_1_ to its less toxic metabolite the hydrolyzed FB_1_ in the gastrointestinal tract of turkeys and pigs [175]. Within 2 h of incubation with FumD, FB_1_ was completely degraded to hydrolyzed FB_1_ in the duodenum and jejunum in an ex vivo pig model [175].

#### Nutritional strategies

It is well accepted that none of the physical, chemical or biological strategies can totally decontaminate the mycotoxin in feed, considering that even a low consumption level of a mycotoxin can cause chronic toxicity including a reduction of the performance and immunosuppression in animals [45], therefore, development of nutritional strategies to help mitigation of the mycotoxicoses is also important. Some nutritional strategies that have been disclosed are presented in Table 6.

**Table 6:** Nutritional strategies to mitigate mycotoxins toxicity^a^

Mycotoxins	Nutritional strategies	Mechanisms	Reference
AFB_1_	Selenium, vitamins C, vitamins E, vitamin B_1_, carotenoids, silymarin, curcumin, butylated hydroxytoluene, alpha lipoic acid, quercetin, resveratrol, rhamnoides oil	Mainly by improving antioxidant capacity and detoxification enzyme activities to alleviate the harm of AFB_1_ to livestock and poultry	[11, 178–188]
DON	Selenium, vitamins C, vitamins E, silymarin, curcumin, functional amino acid (methionine, glutamic acid, arginine, aspartate and lysine), antimicrobial peptide, astragalus	Primarily through enhancement of antioxidant capacity and immune functions to improve the resistance to DON in livestock and poultry.	[179, 180, 189–194]
ZEN	Retinol, as-corbic acid, alpha-tocopherol, silymarin, soybean isoflavone	Alleviated the toxic effects of ZEN by improving the antioxidant capacity and inhibiting the estrogenic toxicity of ZEN.	[17, 190, 195]
FB_1_	Vitamin E, silymarin, curcumin, soybean isoflavone	Mainly via counteracting the oxidative stress caused by FB_1_ to livestock.	[180, 196, 197]

^a^*AFB*_*1*_ Aflatoxin B_1_, *DON* deoxynivalenol, *ZEN* zearalenone, *FB*_*1*_ fumonisin B_1_

It is feasible to modulate the mycotoxin detoxification system through nutritional measures. On the one hand, detoxification systems in animals including CYP450s, ketoreductase, α-glutathione transferase, etc. can degrade mycotoxins [9, 10]. Therefore, any nutrient that can promote the normal functioning of one of the above detoxification enzyme systems can be used as a nutritional regulator. Glutamate, cysteine and glycine can be used as substrates for the synthesis of glutathione and participate in the detoxification process by forming glutathione. On the other hand, mycotoxins can reduce nutrient uptake, so adding critical nutrients is one of the ways to mitigate the harmful effects of mycotoxins [13–15].

Oxidative stress is an important mechanism of cytotoxicity caused by mycotoxins [9, 10]. Adding antioxidants to mycotoxin-contaminated feed can improve the antioxidant capacity of the organism and increase the resistance of livestock and poultry to mycotoxins. Selenium, some vitamins A, C and E, and their precursors have marked antioxidant properties that act as superoxide anion scavengers. For these reasons, these substances have been investigated as protecting agents against toxic effects of mycotoxins. Selenium is an essential trace element for humans and animals as it plays an important role in antioxidant defense, anticancer, immunity, and detoxification [181, 182]. Previous studies have shown that dietary selenium supplementation can help to protect against AFB_1_-induced hepatotoxicity, immunotoxicity, and genotoxicity in chicks, which is mainly associated with regulation of redox/inflammation/apoptotic signaling and CYP450 isozymes [11]. Selenium has the potential to counteract DON-induced immunosuppression in piglets by increased the expression levels of IL-2, IL-10, IFN-γ, IgG, and IgM mRNA and protein in piglet splenic lymphocyte [190]. Selenium, vitamins C and E could be used as antioxidants to protect the spleen and brain cell membranes from DON toxicity and against DNA damage in liver caused by DON [191]. Nagaraj et al. [183] reported that dietary supplemented vitamin B_1_ reduced the toxicity of fusarium in chicks. Vitamin E supplementation counteracts the adverse impacts of FB_1_ on reproductive hormones, gestation length and milk production in rabbits [196]. Grosse et al. [195] observed that retinol, as-corbic acid and alpha-tocopherol reduced DNA adducts in the kidney and liver of mice exposed to ochratoxin A and ZEN from 70-90%. Carotenoids (carotene and xanthophylls) are excellent antioxidants with antimutagenic and anticarcinogenic properties, which have been demonstrated can inhibit AFB_1_-induced liver DNA damage in rats [178].

Silymarin is a potent antihepatotoxic agent provide protection against the negative effects of AFB_1_ on performance of broiler chicks [184]. Curcumin alleviates AFB_1_ toxicity through downregulating CYP450 enzymes, promoting ATPase activities in chickens [185]. Pretreatment with silymarin, curcumin enhanced the viability of cells exposed to the mycotoxins and attenuated reactive oxygen species formation by DON, partially reduced ROS formation by FB_1_ [180]. Curcumin significantly decreased apoptosis in cells exposed to DON, whereas silymarin was able to prevent apoptosis exposed to FB_1_ and DON in PK-15 cells [180]. Gao et al. [17] reported that dietary silymarin supplementation protected rats from ZEN-induced hepatotoxicity and reproductive toxicity through improvement in the antioxidant capacity and regulation in the genes related to ZEN metabolism, hormone synthesis, protein synthesis, and ABC transporters in the tissues.

Butylated hydroxytoluene, a dietary antioxidant in mammals, has been shown to lessen the toxic effects of AFB_1_ by inducing the activity of glutathione sulfotransferase and inhibiting the activity of cytochrome P450 1A5 [198]. Li et al. [186] reported that alpha lipoic acid improved the growth performance and alleviated the liver damage associated with improved the antioxidant capacity in the broilers exposed to AFB_1_. Quercetin exerted its beneficial effects by depressing the bioactivation of AFB_1_ and counterbalancing its pro-oxidant effects in a bovine mammary epithelial cell line [187]. Resveratrol, a polyphenol derived from red grapes, berries and peanuts, exerted anti-inflammatory and antioxidant effects. Dietary supplementation of resveratrol helped in increasing the activities of the oxidative enzymes and in improving the plasma total antioxidant capacity and total protein in broilers fed with AFB_1_ [188]. Solcan et al. [199] reported that *rhamnoides* oil had a potent hepatoprotective activity, reduced the concentration of AFs in the liver and diminished their adverse effects in broilers.

Andretta et al. [192] suggested that methionine can alleviate the DON induced adverse effects in growing pigs. Supplementing glutamic acid, arginine, aspartate and lysine to a diet had positive effects on remission of visceral disease induced by DON, enhancement of antioxidant ability and improvement of blood physiological and biochemical indexes of fattening pigs [200]. Dietary supplementation of 2.0% glutamic acid could mitigate DON induced negative effects on the growth performance and intestinal injury in the weaned piglets [193]. Xiao et al. [179, 194] found that an antimicrobial peptide complex composed of lactoferrin peptide, plant defensin and active yeast effectively improved the adverse effects of DON on production performance, autoimmunity and intestinal functions of weaned piglets. Astragalus played an important role in the reduction of immunosuppression and organ damages of the liver and kidney induced by DON and can improve the immunofunction significantly in mice [189]. Wang et al. [190] suggested that soybean isoflavone added to diets at 600 mg/kg could reduce the harmful effects induced by 2.0 mg/kg ZEN on the reproductive organs in prepubertal gilts during the growth phase. In an in vivo study on rats, Lu [197] reported that soybean isoflavone extract has a marked protective action against FB_1_ hepatotoxicity by the suppression of FB_1_-stimulated prostaglandin production.

## Conclusion and perspectives

The occurrence of mycotoxins in the feed is of a great concern and an unavoidable problem in the feed industry around the world. Mycotoxins also endanger human health through the cycle of the food chain. This review summarizes a number of strategies to reduce mycotoxin contamination in terms of physical detoxification (separation, washing, heating, irradiation and adsorption), chemical treatments (bases and oxidizing agents), biological detoxification methods (microorganisms and enzymes), and nutritional regulation strategies. Each of these approaches can be practically used while along with their own advantages and disadvantages. However, with the growing awareness of environmental protection as well as feed and food safety, there is a growing expectation for more green and innovative technologies to control mycotoxin contamination.

## Data Availability

The datasets used and/or analyzed during the current study are publicly available.

## Abbreviations

AFs: Aflatoxins
AFB_1_: Aflatoxin B_1_
DON: Deoxynivalenol
ZEN: Zearalenone
FB_1_: Fumonisin B_1_
